# Transformed liver cells obtained in culture from hepatectomized rats treated with dimethylnitrosamine (dMN).

**DOI:** 10.1038/bjc.1975.31

**Published:** 1975-02

**Authors:** S. Mondal

## Abstract

**Images:**


*Br. J. Cancer* (1975) **31**, 245

***Short Communication***

### TRANSFORMED LIVER CELLS OBTAINED IN CULTURE FROM HEPATECTOMIZED RATS TREATED WITH DIMETHYLNITROSAMINE (DMN)

S. MONDAL*

*From the Zentrallaboratorium fur Mutagenitätsprüfung, 78 Freiburg i. Br., W. Germany*

Received 10 September 1974. Accepted 15 October 1974

DIMETHYLNITROSAMINE (DMN) is a potent carcinogen and mutagen (Craddock, 1971; Druckrey *et al.*, 1967; Hard and Butler, 1970; Magee and Barnes, 1967; Malling, 1971) and requires metabolic activation in order to exert its harmful effects.

When hamster embryo fibroblast cultures were exposed to DMN, no transformation occurred (Huberman, Salzberg and Sachs, 1968). DiPaolo, Nelson and Donovan (1972) also reported the failure of urethane and diethylnitrosamine added directly to the culture medium to transform the same type of cells. However, transformation did occur in embryo fibroblasts obtained from female hamsters that had been exposed to those compounds during pregnancy. The lack of metabolic competence of fibroblasts to activate this type of chemical oncogen limits their wide use in carcinogenesis tests in culture.

Rat liver is the principal organ that carries out metabolic activation of DMN administered *in vivo* (Magee and Barnes, 1967). Montesano, Saint Vincent and Tomatis (1973) obtained adenocarcinomata when a liver epithelial cell line treated in culture with DMN was transplanted into syngeneic hosts. It is thus evident that the liver epithelium is capable of metabolically activating DMN and similar compounds to yield the ultimate carcinogen and/or mutagen.

However, rat liver cells have been found to undergo spontaneous transformation in culture (Oshiro, Gerscheson and DiPaolo, 1972), and Borek (1972) has reported the transformation of liver epithelium induced by "nutritional stress". The use of an established liver epithelial cell line might therefore invite the criticism that the carcinogen merely accelerated the process of spontaneous transformation. To avoid this drawback, we examined the cells at their earliest stage of development in culture. Furthermore, since carcinogens are known to exert their effect more strongly on dividing cells (Craddock, 1971; Warwick, 1967), we stimulated the liver cells to divide *in vivo* by performing partial hepatectomies on our rats before treatment of animals with DMN, after which we removed the liver and set up the culture.

### MATERIALS AND METHODS

Partial hepatectomies were performed on 2-month-old female BD VI rats (obtained from Professor H. Druckrey of Praeventivmedizin, Freiburg), following the techniques described by Higgins and Anderson (1931). About 20 h after the operation, the rats were injected intraperitoneally with freshly prepared solution of DMN (Aldrich), 50 mg/kg. Forty-eight h after the injection, the rats were killed by cervical dislocation and a specimen from the regenerating liver was taken out aseptically for culture. At the same time a control rat, *i.e.*, a rat that had been hepatectomized on the same day but had received no injection of the oncogen, was also killed and a specimen from the regenerating liver was removed for culture.

* Present address: McArdle Laboratory for Cancer Research, University of Wisconsin Medical Center, Madison, Wisconsin 53706, U.S.A.

The livers were minced very finely and then dissociated progressively at 37°C with 0·1% trypsin and 0·1% collagenase in $Ca^{++}$- and $Mg^{++}$-free phosphate buffer. After dissociation, the trypsin–collagenase solution was removed by centrifugation and the cell pellet was suspended in Hams' $F_{12}$ medium (GIBCO) supplemented with 20% heat-inactivated foetal calf serum (GIBCO) and plated in 60 mm plastic Petri dishes (NUNCLON) at $2{\cdot}5 \times 10^6$ cells/dish in 5 ml of culture medium. The dishes were then incubated in a humidified incubator (Heraeus) at 36·5°C in an atmosphere of 5% $CO_2$ in air. The medium was changed 3 times weekly. In 3 weeks the cells grew as colonies of epithelial-like cells and spindle-shaped fibroblasts. Several colonies of fibroblasts and epithelial-like cells were isolated at random by using siliconized small glass cylinders (Puck, Marcus and Cieciura, 1956) from some dishes of cultures from both treated and untreated rats. At this time other dishes were subcultured in the normal way with 0·1% trypsin in $Ca^{++}$- and $Mg^{++}$-free phosphate buffer solution. All the cells from one dish of primary culture were then replated in 4 dishes. After another week some dishes from this first subculture were again subcultured in the same way. No further subculture was done until the end of the observation period (9 weeks).

### RESULTS

The results described here are from groups of animals (treated and controls) each containing 3 rats. The livers of the treated animals showed widespread degeneration on histological examination.

There were 36 dishes of the first subculture and 48 dishes of the second subculture, in addition to 3 clones of fibroblasts and 3 clones of epithelial-like cells from each group of animals. After 6 weeks from the setting up of the cultures, the first typical transformed foci (1–3/dish) were found in 12 dishes of the first subculture and in 17 dishes of the second subculture of the cells derived from treated animals (Fig. 1, 2). Within another 2 weeks all the dishes of both subcultures of this group of cells showed transformed foci. No such transformed focus was found in any dishes of cells, in both subcultures, derived from control

FIG. 1.—Giemsa stained culture (× 100) of cells from non-treated rat. Cells are gradually dying (7 weeks).

FIG. 2.—Giemsa stained culture (× 100) of cells from DMN-treated rat. Cells are piled up and growing in crisscross patterns (7 weeks).

FIG. 3.—Giemsa stained culture of liver epithelial-like cells (× 40) from non-treated rat (10 weeks).

FIG. 4.—Giemsa stained culture of piled up focus of liver epithelial-like cells (× 40) from DMN treated rat (10 weeks).

animals. All the clones of fibroblasts and epithelial-like cells derived from treated animals also showed transformed foci after 6–12 weeks. A longer time was required for epithelial-like cells to develop the transformed foci. Not a single clone derived from control animals had any such focus during that time. The transformed foci of the epithelial-like cells were distinctly different from the crisscross pattern of transformed fibroblasts (Fig. 3, 4). They started as areas of deeply stained multilayered round cells, gradually spreading and ultimately covering the whole dish with multilayered cells. Both the transformed fibroblasts and epithelial-like cells showed their ability to grow in semi-soft agar (0·33% agar made up with $F_{12}$ medium + 10% foetal calf serum; cloning efficiency was 6–10% when checked after 3 weeks of culture), whereas their control counterparts did not grow. The transformed cells, collected by versene treatment, were agglutinated by concanavalin A (ConA) at a concentration of 50 μg/ml, but the cells from untreated animals did not agglutinate with 250 μg/ml of ConA. Recently, the author has found a similar type of morphologically transformed cells, in culture, from the livers of hepatectomized mice treated with 10 and 5 μg/kg of DMN (Mondal *et al.*, unpublished data).

### DISCUSSION

This work represents an attempt to develop a rapid and convenient test system for oncogenic compounds such as DMN, which is a precarcinogen that requires metabolic activation for its effect. Craddock (1971) obtained carcinomata of the liver in partially hepatectomized rats treated with DMN (9 mg/kg) in a small number of cases (6/22) after a long interval (89–110 weeks), together with sarcomata, lymphosarcomata and kidney tumours. We treated the animals with the test compound and 48 h later removed the liver and set up the cultures without waiting for the development of tumours *in vivo*. We reasoned that, because of the individual immune response, many of the treated animals might not develop a tumour; as a result of the removal of the liver shortly after the treatment, the cells were cut off from the putative immunological defence of the host. This is especially important in the case of compounds having weak carcinogenic activity. By the use of this system, we expected that when the liver cells in the rat are affected by the ultimate carcinogen the cultured cells would then show the changes characteristic of transformation. After hepatectomy, regeneration of all types of cells starts within the latter part of the first day (Higgins and Anderson, 1931), and when a compound is injected at this time its metabolites could be expected to act on the dividing cells without any selection. The regeneration was left, after injection, to continue for another 48 h in order to have the possible transformed state "fixed" by cell division (Borek and Sachs, 1967) and to obtain more mitotic cells that might help in establishing the cultures. In these experiments it was found that setting up the culture from regenerating liver was comparatively easy. Since the cells in this case were at their early stage of culture (only up to the 1st and 2nd subcultures), the chance of selection was also minimized compared with an established cell line after a long period of culture. For these reasons the transformation found in the cells of DMN-treated animals can be attributed to the carcinogenic effect of DMN. Therefore, this combined *in vivo/in vitro* technique may provide a rapid method for testing the oncogenic activity of compounds that need metabolic activation.

The author expresses his sincere thanks to Professor Charles Heidelberger, American Cancer Society Professor of Oncology, McArdle Laboratory for Cancer Research, University of Wisconsin Medical Center, Madison, (U.S.A.) for his help with the manuscript and to Misses Sigrid Niehoff and Ingrid Laass for their skilled technical assistance.

### REFERENCES

BOREK, C. & SACHS, L. (1967) Cell Susceptibility to Transformation by Irradiation and Fixation of the Transformed State. *Proc. natn. Acad. Sci. U.S.A.*, **57**, 1522.

BOREK, C. (1972) Neoplastic Transformation *in vitro* of a Clone of Adult Liver Epithelial Cells into Differentiated Hepatoma-like Cells under Conditions of Nutritional Stress. *Proc. natn. Acad. Sci. U.S.A.*, **69**, 956.

CRADDOCK, V. M. (1971) Liver Carcinomas Induced in Rats by Single Administration of Dimethylnitrosamine after Partial Hepatectomy. *J. natn. Cancer Inst.*, **47**, 899.

DIPAOLO, J. A., NELSON, R. L. & DONOVAN, P. J. (1972) *In vitro* Transformation of Syrian Hamster Embryo Cells by Diverse Chemical Carcinogens. *Nature, Lond.*, **235**, 278.

DRUCKREY, H., PREUSSMANN, R., IVANKOVIC, S. & SCHMÄHL, D. (1967) Organotrope carcinogene Wirkungen bei 65 verschiedenen N-Nitrosoverbindungen an BD-Ratten. *Z. Krebsforsch.*, **69**, 103.

HARD, G. C. & BUTLER, W. H. (1970) Cellular Analysis of Renal Neoplasia: Light Microscope Study of the Development of Interstitial Lesions Induced in the Rat Kidney by a Single Carcinogenic Dose of Dimethylnitrosamine. *Cancer Res.*, **30**, 2806.

HIGGINS, G. M. & ANDERSON, R. M. (1931) Restoration of the Liver of the White Rat following Partial Surgical Removal. *Archs Path.*, **12**, 186.

HUBERMAN, E., SALZBERG, S. & SACHS, L. (1968) The *in vitro* Induction of an Increase in Cell Multiplication and Cellular Life Span by the Water Soluble Carcinogen, Dimethylnitrosamine. *Proc. natn. Acad. Sci. U.S.A.*, **59**, 77.

MAGEE, P. N. & BARNES, J. M. (1967) Carcinogenic Nitroso Compounds. *Adv. Cancer Res.*, **10**, 163.

MALLING, H. V. (1971) Dimethylnitrosamine: Formation of Mutagenic Compounds by Interaction with Mouse Liver Microsomes. *Mutation Res.*, **13**, 425.

MONTESANO, R., SAINT VINCENT, L. & TOMATIS, L. (1973) Malignant Transformation *in vitro* of Rat Liver Cells by Dimethylnitrosamine and N-methyl-N-nitro-N-nitrosoguanidine. *Br. J. Cancer*, **28**, 215.

OSHIRO, Y., GERSCHESON, L. E. & DIPAOLO, J. A. (1972) Carcinomas from Rat Liver Cells Transformed Spontaneously in Culture. *Cancer Res.*, **32**, 877.

PUCK, T. T., MARCUS, P. I. & CIECIURA, S. J. (1956) Clonal Growth of Mammalian Cells *in vitro*, Growth Characteristics of Colonies from Single HeLa Cells With and Without "Feeder Layer". *J. exp. Med.*, **103**, 273.

WARWICK, G. P. (1967) Covalent Binding of Metabolites of Tritiated 2-methyl-4-dimethylaminobenzene to Rat Liver Nucleic Acids and Proteins and the Carcinogenicity of the Unlabelled Compound in Partially Hepatectomized Rats. *Eur. J. Cancer*, **3**, 227.


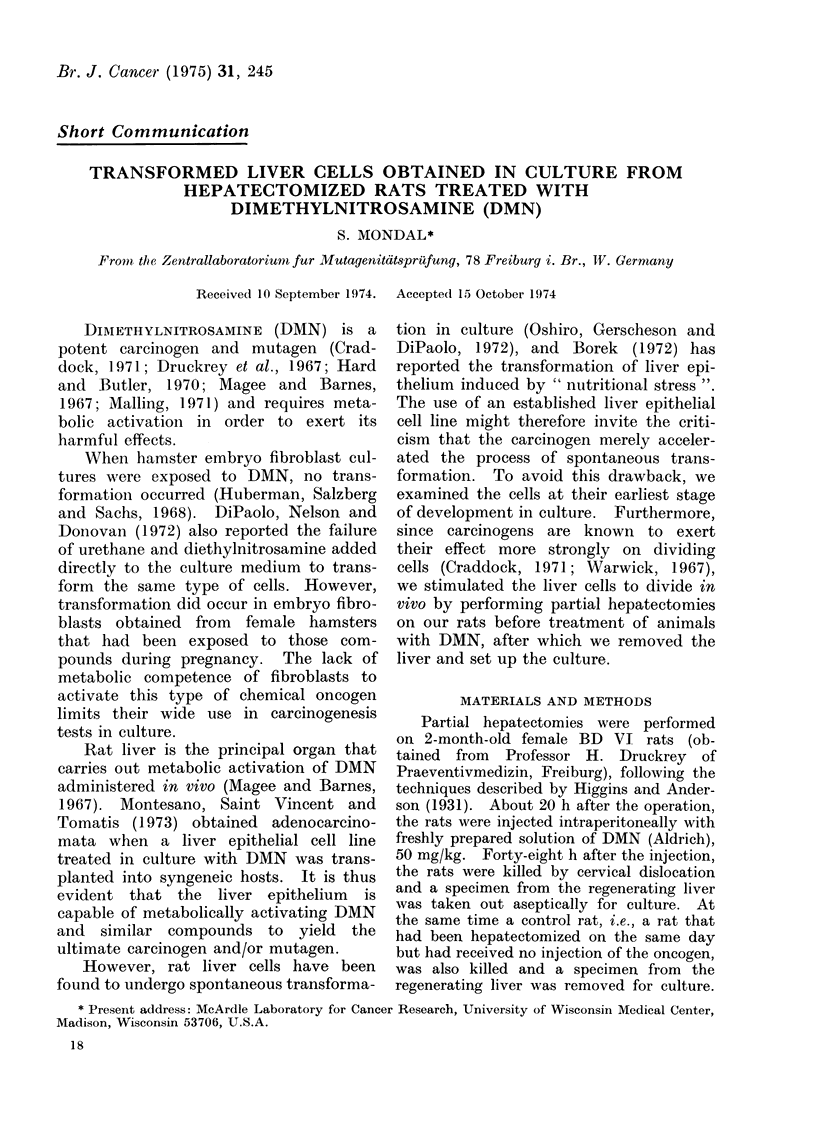

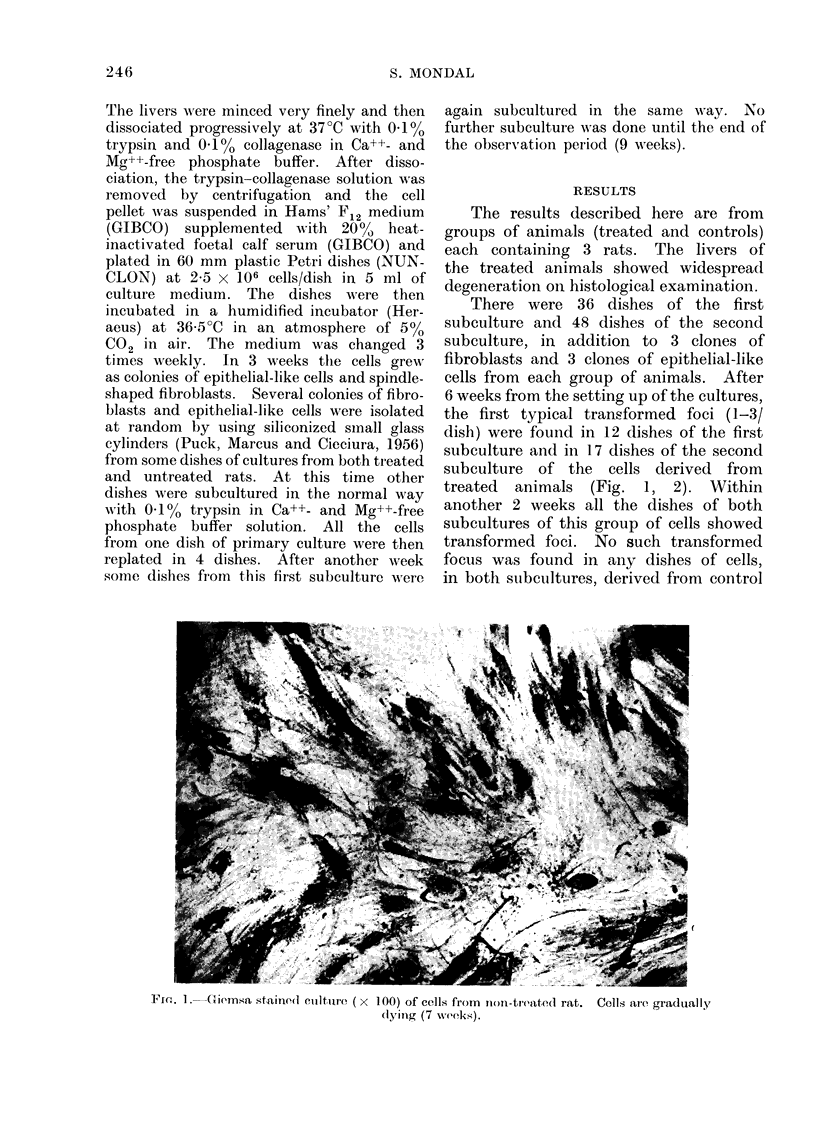

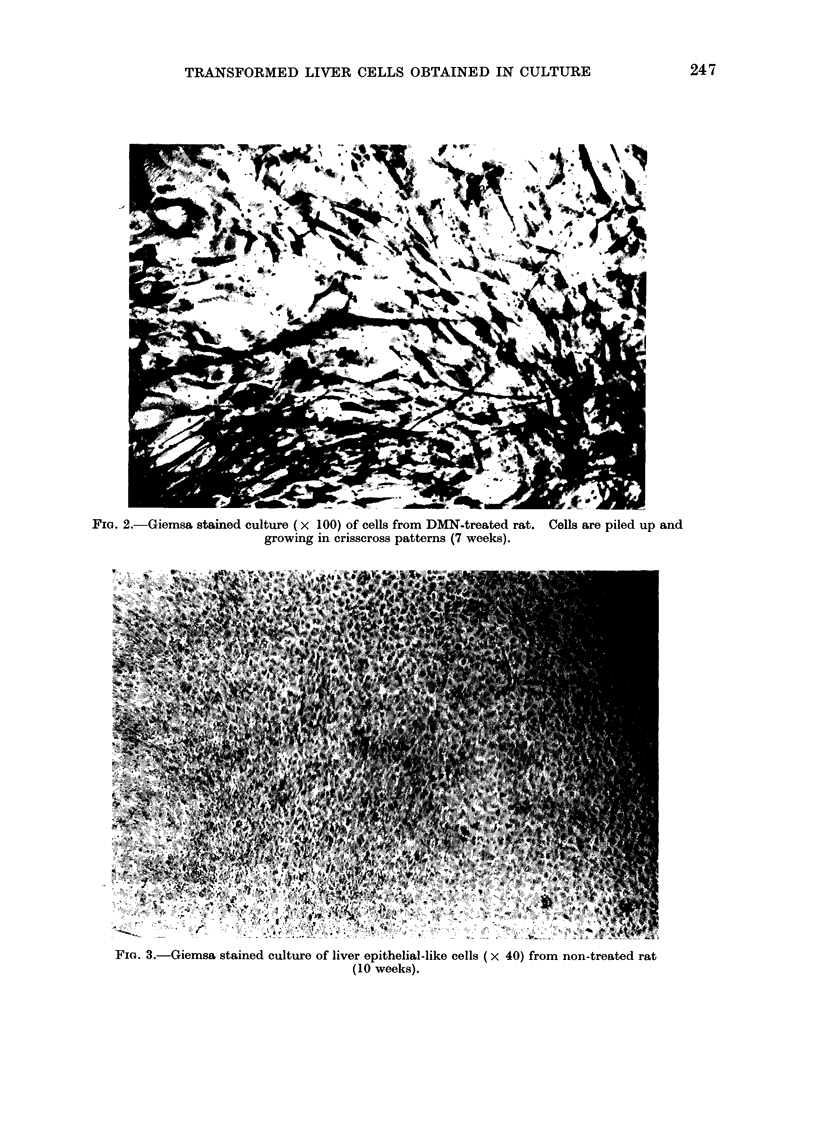

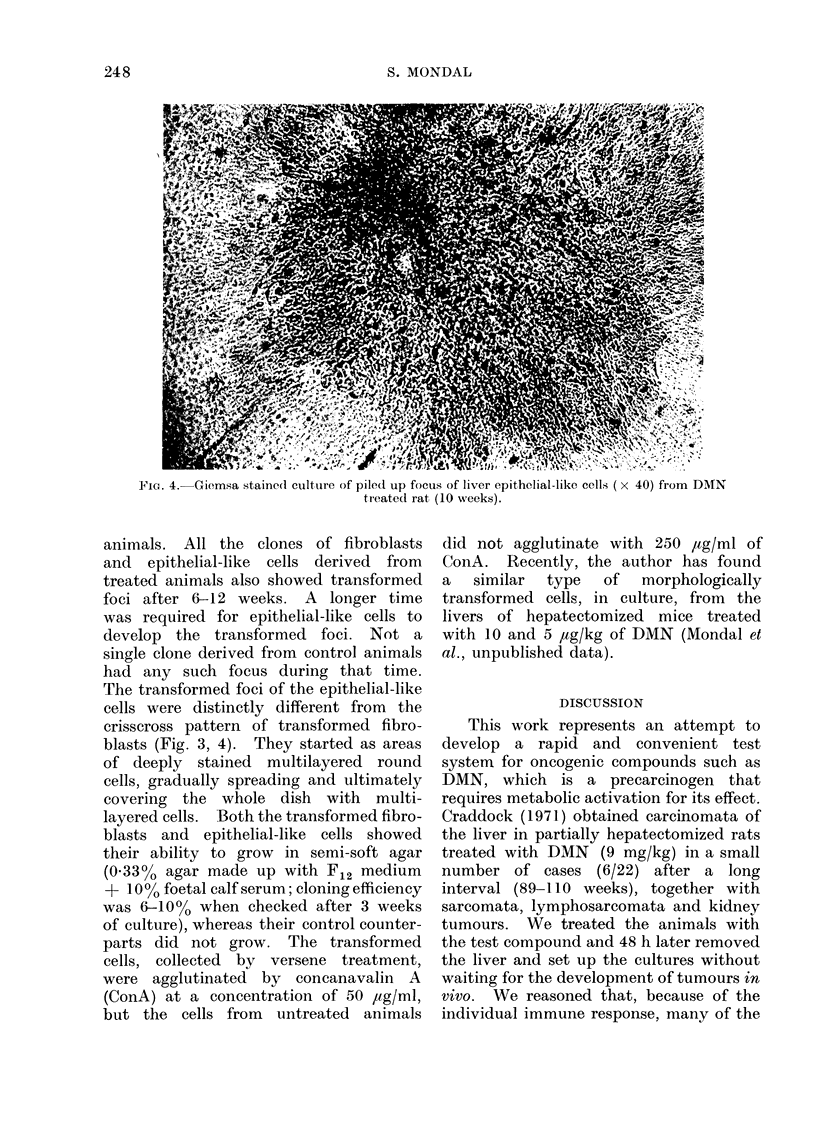

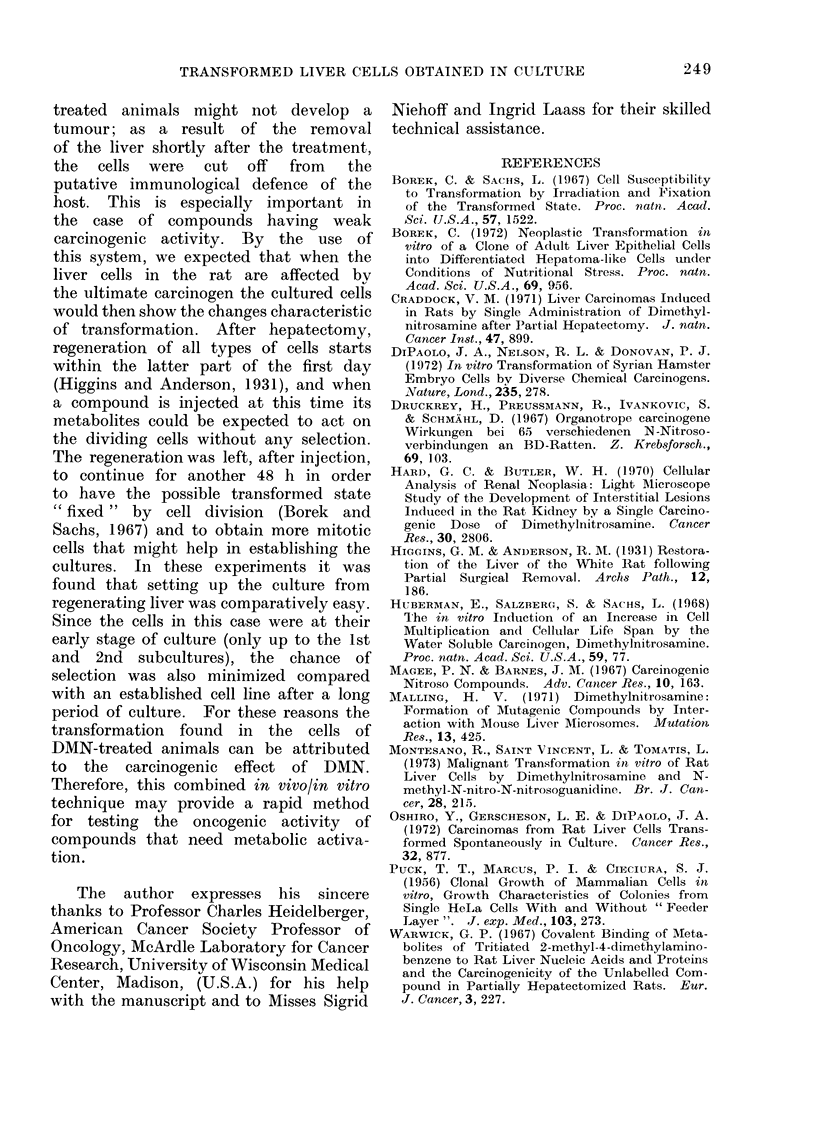


## References

[OCR_00313] Borek C. (1972). Neoplastic transformation in vitro of a clone of adult liver epithelial cells into differentiated hepatoma-like cells under conditions of nutritional stress.. Proc Natl Acad Sci U S A.

[OCR_00307] Borek C., Sachs L. (1967). Cell susceptibility to transformation by x-irradiation and fixation of the transformed state.. Proc Natl Acad Sci U S A.

[OCR_00320] Craddock V. M. (1971). Liver carcinomas induced in rats by single administration of dimethylnitrosamine after partial hepatectomy.. J Natl Cancer Inst.

[OCR_00326] DiPaolo J. A., Nelson R. L., Donovan P. J. (1972). In vitro transformation of Syrian hamster embryo cells by diverse chemical carcinogens.. Nature.

[OCR_00332] Druckrey H., Preussmann R., Ivankovic S., Schmähl D. (1967). Organotrope carcinogene Wirkungen bei 65 verschiedenen N-Nitroso-Verbindungen an BD-Ratten.. Z Krebsforsch.

[OCR_00339] Hard G. C., Butler W. H. (1970). Cellular analysis of renal neoplasia: light microscope study of the development of interstitial lesions induced in the rat kidney by a single carcinogenic dose of dimethylnitrosamine.. Cancer Res.

[OCR_00360] Magee P. N., Barnes J. M. (1967). Carcinogenic nitroso compounds.. Adv Cancer Res.

[OCR_00364] Malling H. V. (1971). Dimethylnitrosamine: formation of mutagenic compounds by interaction with mouse liver microsomes.. Mutat Res.

[OCR_00370] Montesano R., Saint Vincent L., Tomatis L. (1973). Malignant transformation in vitro of rat liver cells by dimethylnitrosamine and N-methyl-N'-nitro-N-nitrosoguanidine.. Br J Cancer.

[OCR_00377] Oshiro Y., Gerschenson L. E., DiPaolo J. A. (1972). Carcinomas from rat liver cells transformed spontaneously in culture.. Cancer Res.

[OCR_00383] PUCK T. T., MARCUS P. I., CIECIURA S. J. (1956). Clonal growth of mammalian cells in vitro; growth characteristics of colonies from single HeLa cells with and without a feeder layer.. J Exp Med.

[OCR_00390] Warwick G. P. (1967). The covalent binding of metabolites of tritiated 2-methyl-4-dimethylamino-azobenzene to rat liver nucleic acids and proteins, and the carcinogenicity of the unlabelled compound in partially hepatectomised rats.. Eur J Cancer.

